# Development of gluten‐free bread formulations containing whole chia flour with acceptable sensory properties

**DOI:** 10.1002/fsn3.495

**Published:** 2017-06-26

**Authors:** Luciana T. B. Sandri, Fernanda G. Santos, Camilly Fratelli, Vanessa D. Capriles

**Affiliations:** ^1^ Departamento de Biociências campus Baixada Santista Universidade Federal de São Paulo Santos São Paulo Brazil

**Keywords:** Chia seed, Mixture design, Physicochemical properties, Sensory analysis

## Abstract

Increasing the variety of better‐tasting and healthier gluten‐free products is important for consumers with gluten‐related disorders. This work aimed to develop a gluten‐free bread formulation containing whole chia flour with acceptable sensory properties. A mixture design for three ingredients and response surface methodology were used to identify the proportions of potato starch, rice flour and whole chia flour to achieve the best physical properties and result in sensory‐accepted products. The physical properties and visual appearance showed that whole chia flour alone is not suitable for bread production. Nevertheless, it is possible to add up to 14% whole chia flour to a rice flour‐based gluten‐free bread formulation while negligibly diminishing the loaf volume, crumb firmness and crumb moisture. The best formulations were prepared from rice flour blends with 5, 10, and 14% whole chia flour, which received overall acceptability scores of 8.7, 8.1 and 7.9 on a 10‐cm scale, respectively, similar to those of their white gluten‐free bread and wheat bread counterparts. Incorporating 5%–14% whole chia flour in the formulation increased the levels of ash, lipid, protein and dietary fiber compared to those of the white gluten‐free bread.

## 1 INTRODUCTION

Despite the considerable advances in gluten‐free (GF) research and the impressive growth of the GF market in recent years, individuals with gluten‐related disorders still have trouble finding GF products because of high prices, limited variety and availability and poor sensory properties. These factors are responsible for hampering adherence to the GF diet and for general dissatisfaction (do Nascimento, Fiates, dos Anjos, & Teixeira, 2014).

The development of GF products remains a technological challenge due to the role of gluten in various cereal‐based products, especially in bread and pasta making. Amongst all the gluten‐free products, bread is the most globally studied (Capriles, Santos, Reis, & Pereira, 2015). However, a gluten‐free bread (GFB) with a good sensory aspect is still the most desired product by individuals with gluten‐related disorders, such as celiac disease (do Nascimento, Fiates, dos Anjos, & Teixeira, 2014).

A range of GFB formulations have been developed using rice and maize flours, which are often combined with maize, potato, or cassava starches as base flours because they are widely available, inexpensive ingredients that are bland in taste and flavor. However, these GF flours and starches are not generally enriched or fortified and neither are the resultant GFBs, unlike their wheat‐based counterparts (do Nascimento, Fiates, dos Anjos, & Teixeira, 2013; Kinsey, Burden, & Bannerman, 2008; Thompson, 2000; Thompson, Dennis, Higgins, Lee, & Sharrett, 2005). Therefore, such products may lead to nutritional deficiencies in individuals who face the daily challenges imposed by a strict gluten‐free diet (Capriles, Santos, & Arêas, 2016). Thus, more research and development are required to increase the variety of better‐tasting and healthier GF products. This can be done by incorporating natural raw materials rich in nutrients and bioactive compounds, such as chia seed, into GFB formulations (Capriles, Santos, & Arêas, 2016; Torres, Arufe, Chenlo, & Moreira, 2017).

The chia seed (*Salvia hispanica* L.) was an important staple food for pre‐Columbian societies in Central America. Following the recent evaluation of their nutritional and functional potential, chia seeds have attracted a great deal of interest in the research community and food and pharmaceutical industries, as well as among consumers (Munoz, Cobos, Diaz, & Aguilera, 2013). The chia seed has been described as a good source of protein (18%–25%), dietary fiber (20%–37%) and oil (21%–33%), of which approximately 60%–63% is α‐linolenic acid (Munoz, Cobos, Diaz, & Aguilera, 2013; Porras‐Loaiza, Jimenez‐Munguia, Sosa‐Morales, Palou, & Lopez‐Malo, 2014). In addition, the chia seed is rich in phenolic compounds and has high in vitro antioxidant activity (Marineli et al., 2014; Porras‐Loaiza, Jimenez‐Munguia, Sosa‐Morales, Palou, & Lopez‐Malo, 2014).

Thus far, few studies have been performed on the use of chia seeds in GF bread‐making. Moreira, Chenlo, and Torres (2012, 2013) incorporated 2.5%–7.5% whole chia flour (WCF) into a gluten‐free chestnut flour‐based dough. These authors concluded that the addition of 7.5% WCF improved the dough rheological properties of stability, viscosity and elasticity. Costantini et al. (2014) replaced common and tartary buckwheat flour with 10% WCF, and they observed an improvement in the protein, lipid, dietary fiber, ash, α‐linolenic acid, and phenolic compound contents as well as in the antioxidant capacity of the formulations. Steffolani, de la Hera, Perez, and Gomez (2014) observed that the replacement of rice flour with 15% WCF or 15% chia seeds darkened the GFB, reduced the specific volume, and increased the hardness, but it does not reduce the overall acceptability (for scores of approximately 5 ‐ neither like nor dislike, on a 9‐point hedonic scale). Huerta, Alves, Silva, Kubota, and Rosa (2016) replaced rice and soy flour with 2.5, 5.0 and 7.5% WCF, and they observed that bread with 2.5% WCF showed no significant differences in relation to the control for the specific volume and baking loss as well as for the color, aroma, taste, texture, and appearance acceptability (scores ranging from 4.5 to 5.5, on a 7‐point hedonic scale).

These studies showed the potential use of WCF in GFB, but to the best of our knowledge, there have been no reports to date on the optimization of the WCF proportions in GFB formulations. Considering that, the objective of this study was to use a mixture design to define the optimum WCF proportions in a GFB formulation with acceptable sensory properties. A mixture design for three ingredients and response surface methodology were used to identify the proportions of WCF in various blends with potato starch (PS) and rice flour (RF) achieving the best physical properties. Subsequently, the physical properties and sensory acceptability scores of the best formulations were compared to those of their white GFB and wheat bread counterparts.

## 2 MATERIALS AND METHODS

### 2.1 Materials

Chia seed (Benexia^®^ Chia omega‐3) from Santa Cruz Valley, Bolivia was supplied by R & S Blumos Comercial de Produtos Alimentícios Ltda (São Paulo, Brazil). Whole chia flour was obtained by grinding the seeds into flour with a coffee grinder (MDR301, Cadence Indústria e Comércio Ltda., Brazil). The resulting flour was packed in polyethylene bags and stored at room temperature (approximately 25°C) prior to further use.

Xanthan gum (Ziboxan F80, Deosen Biochemical Ltd, China) and carboxymethylcellulose (DENVERCEL FG‐2504A, Denver Especialidades Químicas Ltda, Brazil) were donated by Eurogerm Brasil Produtos Alimentícios Ltda. (São Paulo, Brazil). The other ingredients were obtained at the local market.

The flour/starch blends consisted of WCF, RF, and PS. The compositions were determined in triplicate by standard methods (AOAC 2005). The results for WCF were 4.5% ash, 29.9% total lipids, 23.4% protein, 42.2% dietary fiber, and 0.0% available carbohydrate dry weight (dw). The RF had 0.5% ash, 0.9% total lipids, 8.4% protein, 4.6% dietary fiber, and 85.5% available carbohydrate dw. The PS had an available carbohydrate content of 99.1% dw and presented no significant amounts of ash, fat, protein, or dietary fiber.

The particle size distributions were 92% 425 μm and 8% 250 μm for WCF; 24% 425 μm, 46% 250 μm, 28% 180 μm, and 2% ≤ 150 μm for RF; and 4% 425 μm, 29% 250 μm, 31% 180 μm and 36% ≤ 150 μm for PS. The particle size distributions were determined according to AOAC method 965.22 (AOAC 2005).

### 2.2 Gluten‐free bread preparation

The GFB formulation consisted of the following, on a % of total flour weight basis (fwb): 100% flour/starch blend, 100% water, 25% whole egg, 10.5% whole milk powder, 6% white cane sugar, 6% soy oil, 2% salt, 0.8% instant dry yeast, 0.3% xanthan gum, and 0.3% carboxymethylcellulose. The flour/starch blend consisted of RF, PS, and WCF in blends summing to 100% (fwb), according to the experimental design.

A straight dough process was performed using a stand mixer (BPS‐05‐NSkymsen, Metalúrgica Siemsen Ltda., Brazil) with a paddle attachment. All ingredients were mixed at speed 4 (on a 1–10 mixer scale) for 4 min. The resulting dough (400 g) was then spread into previously greased and floured baking pans (19 × 7.5 × 5 cm) and proofed in a proofing chamber at 40°C and 85% relative humidity for 45 min (CFK‐10, Klimaquip S/A – Tecnologia do Frio, Brazil). Baking was performed in an electric oven at 160°C for 22 min (HPE‐80, Prática Produtos S.A., Brazil). After baking, the loaves were depanned and cooled for 2 hr on cooling racks at room temperature. The loaves were then stored in polyethylene bags to prevent moisture loss at room temperature (approximately 25°C). All analyses were performed within 3 hr.

Six loaves for each of the GFB trials were prepared from one batch. Three random loaves were used for the specific volume and crumb moisture analyses, and three random loaves were used for the crumb texture evaluation and photographs. An extra six loaves from the selected treatments were produced for sensory evaluation.

### 2.3 Experimental design

The simplex‐centroid design for mixtures of three ingredients was used to study the effects of pure and binary and tertiary mixtures of RF (x_1_), PS (x_2_), and WCF (x_3_) on the physical properties of GFB. The experiment was performed on three centroid point replications and included three axial points, for a total of twelve trials (Table 1), prepared using a previously randomized execution sequence (Cornell, 2002). The flour/starch blend corresponded to 35.8% of the dough for all formulations.

**Table 1:** Mixture experimental design and physical properties of gluten‐free bread formulations

Trial	Component proportion in flour/starch blend^a^			Bake loss (%)^b^	Loaf specific volume (cm^3^/g)^b^	Crumb firmness (*N*)^b^	Crumb moisture (%)^b^
	x_1_	x_2_	x_3_				
1	1.00	0.00	0.00	8.17^ab^ ± 0.41	1.69^a^ ± 0.01	15.22^de^ ± 0.49	52.08^ef^ ± 0.06
2	0.00	1.00	0.00	8.06^abc^ ± 0.61	1.38^bc^ ± 0.04	14.98^de^ ± 1.77	55.17^a^ ± 0.18
3	0.00	0.00	1.00	7.27^abc^ ± 0.47	1.22^d^ ± 0.02	31.87^a^ ± 4.02	51.41^f^ ± 0.75
4	0.50	0.50	0.00	8.42^a^ ± 0.56	1.70^a^ ± 0.02	8.92^e^ ± 1.37	53.26^bc^ ± 0.17
5	0.50	0.00	0.50	6.86^bc^ ± 0.49	1.43^bc^ ± 0.02	29.11^ab^ ± 1.27	51.38^f^ ± 0.03
6	0.00	0.50	0.50	6.98^abc^ ± 0.15	1.31^cd^ ± 0.03	23.81^bc^ ± 3.33	53.45^bc^ ± 0.23
7	0.33	0.33	0.33	6.65^c^ ± 0.33	1.30^cd^ ± 0.08	31.75^a^ ± 5.00	52.76^cde^ ± 0.25
8	0.33	0.33	0.33	7.54^abc^ ± 0.14	1.29^cd^ ± 0.02	30.27^ab^ ± 6.77	53.05^cd^ ± 0.09
9	0.33	0.33	0.33	7.06^abc^ ± 0.69	1.33^cd^ ± 0.08	31.58^a^ ± 6.28	52.86^cde^ ± 0.25
10	0.66	0.17	0.17	7.52^abc^ ± 0.48	1.52^b^ ± 0.04	24.91^abc^ ± 1.18	52.24^de^ ± 0.29
11	0.17	0.66	0.17	7.81^abc^ ± 0.62	1.51^b^ ± 0.08	21.08^cd^ ± 4.12	53.93^b^ ± 0.11
12	0.17	0.17	0.66	7.06^abc^ ± 0.69	1.33^cd^ ± 0.08	28.24^abc^ ± 1.67	52.12^ef^ ± 0.09

a x_1_= Rice flour, x_2_= Potato starch, x_3_= Whole chia flour.

b Values are means ± standard deviations. Values followed by different superscripts in each row are significantly different (*p *<* *.05).

### 2.4 Physical property evaluations

After cooling, the loaves were weighed (UX‐6200H, Shimadzu Corporation, Japan), and the loaf volumes were measured by millet‐seed displacement (Vondel Indústria e Comércio de Máquinas e Componentes EIRELI – ME., Brazil) according to AACC method 10‐05.01 (AACC 2010). The loaf‐specific volume (volume [cm^3^]/ weight [g]) and the bake loss were also evaluated in triplicate.

The crumb moisture was evaluated in triplicate, according to AACC method 44‐15A (AACC 2010). The crumb firmness was determined according to AACC method 74‐09 (AACC 2010) using a texture analyser (TA.XT*plus*, Stable Micro Systems, Surrey, UK). Texture measurements (six values) were performed on two bread slices that were taken from the centers of three different loaves.

### 2.5 Physical property optimization and quality verification

The bread physical properties were used as response variables for the mixture design regression models. The following Scheffé canonical polynomial models were applied:

$$\text{Linear equation}:Y=\beta_{1}x_{1}+\beta_{2}x_{2}+\beta_{3}x_{3}$$

$$\text{Quadratic equation}:Y=\beta_{1}x_{1}+\beta_{2}x_{2}+\beta_{3}x_{3}+\beta_{12}x_{1}x_{2}+\beta_{13}x_{1}x_{3}+\beta_{23}x_{2}x_{3}$$

$$\begin{array}{cc} \text{Special cubic equation}:Y= & \beta_{1}x_{1}+\beta_{2}x_{2}+\beta_{3}x_{3}+\beta_{12}x_{1}x_{2}+\beta_{13}x_{1}x_{3}\\ & +\beta_{23}x_{2}x_{3}+\beta_{123}x_{1}x_{2}x_{3} \end{array},$$

where Y is the response variable; β_1_, β_2_, β_3_, β_12_, β_13_, β_23_, and β_123_ are regression parameters; and x_1_, x_2_, and x_3_ are the proportions of RF, PS, and WCF, respectively, in the flour/starch blend.

Each response in the linear model represents the effects of a pure ingredient. The quadratic model adds the effects of the binary mixtures, and the special cubic model includes the effects of the ternary blends. Positive values for the binary coefficients (β_12_, β_13,_ and β_23_) or the ternary coefficient (β_123_) indicate synergistic effects, and negative values represent antagonistic effects between the ingredients (Cornell, 2002). Based on the regression model significance, contour plots were then produced to determine the optimal blend regions, and the best formulations were properly selected to achieve the best physical properties. These GFBs were prepared and experimentally analyzed, and the results were statistically compared to the predicted values from the fitted models.

### 2.6 Sensory evaluation for acceptance

The sensory acceptability of the selected GFB formulations was evaluated by 50 untrained panellists (32 females and 18 males, aged 19–54 years) recruited from the campus via internal announcements. All the panelists agreed to taste the samples before the tests occurred and attested that they had bread‐consuming habits and did not have any allergy or intolerance to any of the ingredients present in the products. They had no gluten‐related disease and were made aware that they were tasting GFBs. The Human Research Ethics Committee of the Federal University of São Paulo approved the research protocol under number 203.145. Informed consent was obtained from all individuals in the study.

The panellists scored the appearance, color, aroma, texture, taste, and overall acceptability of the formulations on a 10‐cm hybrid hedonic scale (Villanueva, Petenate, & Da Silva, 2005). The bread slices (12.5 mm thick) were separately offered in a random sequence in polyethylene bags coded with 3‐digit numbers. The evaluation was conducted in a climate‐controlled (20°C–25°C) sensory evaluation laboratory equipped with separate booths. The panellists rinsed their mouths with water between samples to minimize any residual effects.

### 2.7 Proximate composition

The proximate compositions of the selected GFB formulations were determined according to the AOAC methods (AOAC, 2005). The moisture content was calculated based on weight loss after the sample was heated in an oven at 105°C. Ash content was determined by incineration in muffle furnace at 550°C. Protein content was determined by total nitrogen, obtained by micro‐Kjeldahl, considering conversion factor of %N × 6.25. Fat was determined by the Soxhlet method. Total dietary fiber by enzymatic–gravimetric, using a commercial assay kit (K‐TDFR, Megazyme International Ireland Limited, Bray, Ireland). Available carbohydrates were calculated by difference [100 ‐ (moisture + ash + protein + fat + dietary fiber)]. Each value was the average of three determinations.

### 2.8 Statistical analysis

Differences in treatment means were identified by one‐way analysis of variance (ANOVA) and Tukey's test. A simple linear correlation (Pearson correlation coefficient) was also evaluated. The model adequacies were checked by variance analysis (*F* test), R^2^ values, lack‐of‐fit tests, and diagnostic plots such as normal and residual plots. Data were processed using Statistica 12.0 statistical software (StatSoft Inc., Tulsa, OK, USA, 2013).

## 3 RESULTS AND DISCUSSION

Table 1 shows the physical properties of the twelve experimental GFB formulations. The results show that the higher the WCF proportion in the flour/starch blend, the lower the loaf specific volume (*r* = −.73, *p* < .01) and the higher the crumb firmness (*r* = .73, *p* < .01). These technological limitations of WCF use were also observed by Pizarro, Almeida, Samman, and Chang (2013), who added 0%–30% WCF to a wheat‐based pound cake, by Steffolani et al. (2014), who added 15% WCF to a rice‐based GFB, and by Coelho and Salas‐Mellado (2015), who added 2%–20% WCF to wheat bread.

This negative effect of WCF on the GFB′s physical properties could be a consequence of the WCF particle size distribution and composition and also the hydration level effects on the dough properties. The coarse WCF with bran particles probably disrupted the gas cells and starch gel uniformity in the dough, resulting in bread with a low specific volume and crumb softness. The formulation prepared with 66%–100% WCF (trials 3 and 12, Table 1) presented a higher dough consistency, making it difficult to mix and then incorporate gas cells during the mixing step. These effects likely occur because of the chia protein, dietary fiber, and mucilage water‐binding capacity, and the starch dilution effect. These factors could limit starch swelling and gelatinization, which together with the bran particle effects impaired the GFB expansion, structure and texture (Capriles & Arêas, 2014). Additionally, the high levels of fat present in the WCF may have implications for the GF dough and bread properties.

The water levels were fixed during this mixture design study. This variable could impair the GF dough and bread properties because increasing the amount of water is usually necessary in formulations that are enriched with fiber or fiber‐rich flours. Increasing the amount of water allows for the adequate dough viscosity, starch gelatinization, and protein denaturation required during bread‐making (Capriles & Arêas, 2014). Further studies could evaluate the effects of water level adjustments on chia‐containing GF dough and bread.

**Figure 1:**
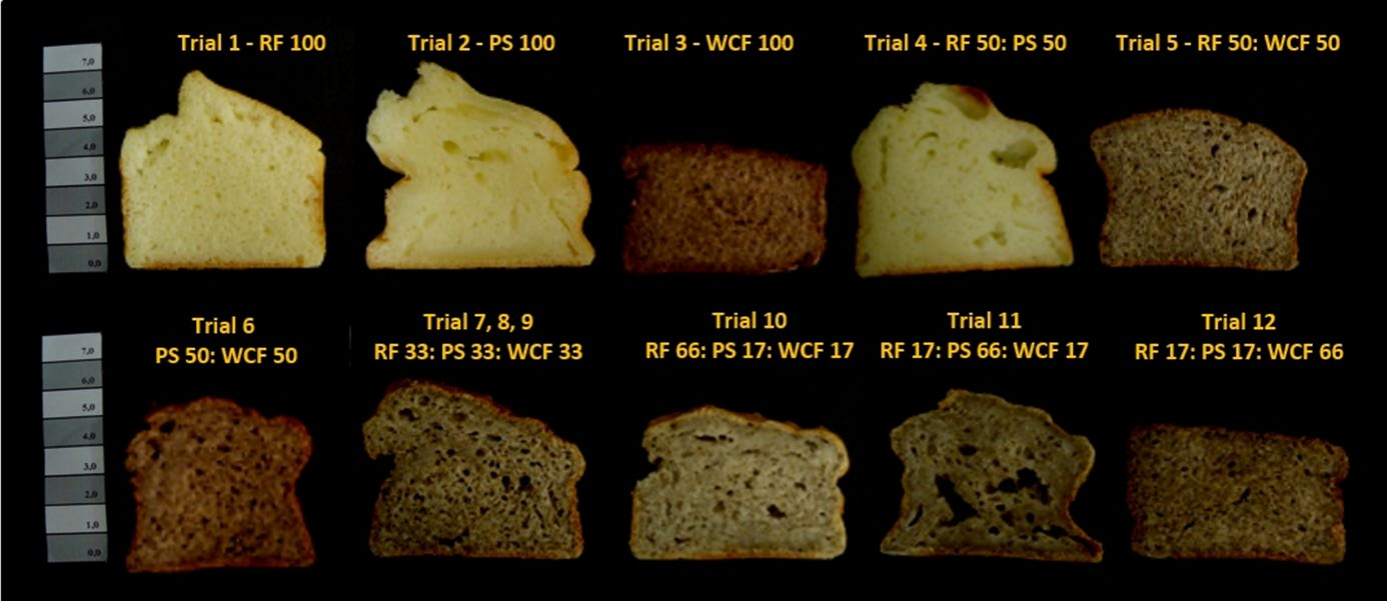
Appearances of central slices of twelve gluten‐free bread formulations obtained from the experimental mixture design. Bread IDs: RF = rice flour, PS = potato starch, WCF = whole chia flour. The numbers indicate the ingredient proportions on a flour weight basis (g/100 g)

It is possible to prepare a GFB made from 100% WCF. However, as shown in Figure 1 and Table 1, it is clear that using 100% WCF impaired the structure, texture, appearance, and color of GFB, and it also presented a poor mouthfeel and flavor. Similar technological limitations related to the use of whole‐grain flour in GFB were reported by some researchers, including changes in the appearance, color, texture, aroma, and taste, which can easily impair consumer acceptability (Hager et al., 2012; Onyango, Unbehend, & Lindhauer, 2009; Schober, Messerschmidt, Bean, Park, & Arendt, 2005). Because of its own gray color, WCF darkened the GFB crumb. This darkening effect was also reported in other studies on baked products (Coelho & Salas‐Mellado, 2015; Costantini et al., 2014; Pizarro et al., 2013; Steffolani et al., 2014).

The physical properties and visual appearance show that WCF alone is not suitable for bread production. Nevertheless, it was noted that the 17%–50% WCF blend with RF and PS resulted in a GFB with better physical properties and appearance (Table 1 and Figure 1).

**Figure 2:**
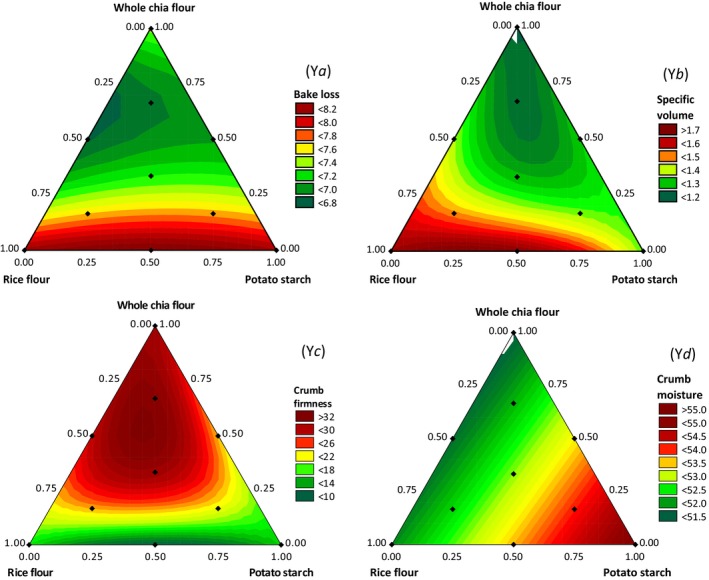
Contour plots for the physical properties of gluten‐free bread based on mixture design regression models. Y*a=* bake loss (%), Y*b*= loaf‐specific volume (cm^3^/g), Y*c=* crumb firmness (N), and Y*d=* crumb moisture (%)

The mixture regression models for the physical properties of GFB are given in Table 2. All the models were significant, and presented no lack of fit and high adjusted coefficients of determination (R^2^_adj_), with 72% to 97% of the variation being explained by the models. These well‐adjusted models were used to generate the contour plots for the physical properties of the GFB (Figure 2).

**Table 2:** Predicted model equations for the mixture design indicating the effect of each mixture component^a^ and their interactions on the physical properties of the gluten‐free bread

Parameter	Predicted model equations^b^	*R* ^2^_adj_ (%)^c^	Model *(p)* d	Lack of fit (*p*)^d^
Bake loss	Y*a* = 8.15RF +8.12PS +7.34WCF ‐3.85RF x WCF	72.5	.019	.938
Loaf specific volume	Y*b* = 1.68RF +1.40PS +1.23WCF ‐4.98RF x PS x WCF	84.8	.009	.068
Crumb firmness	Y*c* = 15.9RF +15.1PS +30.6WCF +283.9RF x PS x WCF	91.0	.003	.072
Crumb moisture	Y*d* = 51.85RF +55.16PS +51.42WCF	97.4	.000	.449

a Mixture components: RF, Rice flour; PS, Potato starch; WCF, Whole chia flour.

b Only the coefficients significant at the *p *<* *.05 level were selected for the predicted model construction.

c *R* ^2^_adj_ adjusted coefficient of determination.

d Significance of the model and Lack of fit. *p * =  probability level.

Figure 2a shows that GFB prepared with higher proportions of RF and PS presented higher bake losses than those made with higher proportions of WCF. Because of antagonistic effects, the GFBs made from blends of RF and WCF present lower bake losses than breads made from the pure ingredients. Steffolani et al. (2014) also observed that the addition of WCF tended to produce a reduction in bake loss, and this effect can be related to a loaf volume with a lower surface area for exchange with the exterior and also to chia mucilage because of its water‐holding capacity.

The loaf‐specific volume was inversely correlated with the crumb firmness (*r* = −0.80, *p* < .01), and thus a lower loaf‐specific volume results in a greater firmness because of the denser crumb and more compact cells. Figure 2b and c show that GFBs containing higher proportions of WCF exhibit a lower loaf volume and higher crumb firmness, while GFBs made with blends of RF and PS exhibit a higher volume and lower crumb firmness.

Figure 2d shows that the crumb moisture only ranged from 51.5% to 55.0%. GFBs made from higher proportions of PS present a higher crumb moisture, and the GFBs containing higher WCF proportions have the lowest values.

From the regression coefficients shown in Table 2, it is clear that the ternary interactions between RF, PS, and WCF are the terms that most affect the loaf volume and crumb firmness. These interactions diminished the GFB quality, reducing the loaf volume and crumb softness. Hence, these results show that GFBs made from RF, PS, and WCF blends do not present good physical properties. Moreover, Figure 2a–d shows that small proportions of WCF do not necessarily have a negative impact on the GFB physical properties, especially when blended with RF.

The focus of this study was to verify the suitability of GFBs containing WCF. Considering that the loaf volume is directly related to the crumb softness and texture acceptance (Capriles & Arêas, 2014), promising formulations were selected considering the models fitted to these physical properties. GFB formulations prepared with blends of RF and WCF were selected from models presenting high loaf volume values and lower crumb firmness values, which could result in sensory‐accepted products. Confirmatory experiments were performed, and the results show that the loaf volume and crumb firmness of GFBs made from RF blends with 5%, 10% and 14% WCF corresponded well with the predicted values. No differences were detected in the loaf volume or crumb firmness between these GFB formulations (Table 3).

**Table 3:** Predicted and measured values for the loaf specific volume and crumb firmness of the optimized gluten‐free bread formulations containing whole chia flour

Trial	Component proportion in flour/starch blends^a^			Predicted values^b^^,^ c		Measured values^c^^,^ d ^,^ e	
	x_1_	x_2_	x_3_	Loaf specific volume (cm^3^/g)	Crumb firmness (*N*)	Loaf specific volume (cm^3^/g)	Crumb firmness (*N*)
A	0.95	0.00	0.05	1.66 (1.54–1.78)	16.57 (12.16–20.99)	1.64^a^ (1.56–1.72)	19.02^a^ (17.26–20.79)
B	0.90	0.00	0.10	1.63 (1.51–1.75)	17.36 (12.94–21.77)	1.72^a^ (1.63–1.81)	15.30^a^ (14.22–15.98)
C	0.86	0.00	0.14	1.62 (1.50–1.74)	17.95 (13.53–22.36)	1.69^a^ (1.66–1.71)	15.89^a^ (14.61–20.79)

a x_1_= Rice flour, x_2_= Potato starch, x_3_= Whole chia flour_._

b Predicted values from fitted models (Table 2).

c Values are the means and 95% confidence intervals.

d Measured values from the confirmatory assay.

e Values followed by different superscripts in each row are significantly different (*p *<* *.05).

The results of the mixture design experiments showed that GFBs with good physical properties could be prepared with 5%, 10% and 14% WCF. These formulations present loaf volumes similar to those of two white GFBs, which were prepared with 100% RF and with 50% RF and 50% PS, but they had slightly higher crumb firmness values (trials 1 and 4 from Table 1).

The GFB formulations made from RF blends with 5%, 10% and 14% WCF were accepted, with scores for appearance, color, aroma, texture, taste, and overall acceptability ranging from 7.3 to 8.7 on a 10‐cm hybrid hedonic scale, as shown in Table 4. However, the GFBs containing 10% and 14% WCF presented darker crust and crumb colors, which diminish the appearance and color acceptability compared with those of the white GFBs that were prepared with 100% RF and with a 50% RF and 50% PS blend (fwb) and received sensory scores ranging from 8.2 to 8.5 according to the results recently reported by Capriles, Santos, and Arêas, (2016). No significant differences were observed between the aroma, texture, taste and overall acceptability scores of the chia‐containing GFB and the white GFB, with scores ranging from 7.6 to 8.2, and neither with the standard wheat bread counterpart (scores ranging from 7.6 to 8.1)(Capriles, Santos, & Arêas, 2016).

**Table 4:** Sensory acceptability scores of optimized gluten‐free bread formulations containing whole chia flour

Trial	Component proportion in flour/starch blends^a^			Acceptability scores^b^					
	x_1_	x_2_	x_3_	Appearance	Color	Aroma	Texture	Taste	Overall
A	0.95	0.00	0.05	8.53^a^ ± 1.60	8.56^a^ ± 1.37	8.70^a^ ± 1.27	8.66^a^ ± 1.15	8.17^a^ ± 1.73	8.65^a^ ± 1.16
B	0.90	0.00	0.10	7.61^b^ ± 1.50	7.79^b^ ± 1.40	8.43^a^ ± 1.59	8.20^a^ ± 1.62	8.00^a^ ± 1.75	8.08^a^ ± 1.52
C	0.86	0.00	0.14	7.53^b^ ± 1.56	7.27^b^ ± 1.74	8.40^a^ ± 1.44	8.08^a^ ± 1.43	7.92^a^ ± 1.52	7.88^b^ ± 1.33

a x_1_= Rice flour, x_2_= Potato starch, x_3_= Whole chia flour_._

b Values are means ± standard deviations (*n* = 50) of acceptability scores on a 10‐cm hybrid hedonic scale. Values followed by different superscripts in each row are significantly different (*p *<* *.05).

The GFB formulations made from RF blends with 5%, 10%, and 14% WCF, for which the proximate compositions are presented in Table 5, presented significant increases in the ash, lipid, protein and dietary fiber contents compared to the white bread. The GFB prepared with 100% RF presented 52.2% moisture, 1.2% ash, 4.1% lipids, 5.0% protein, 2.1% dietary fiber, and 35.4% available carbohydrates.

**Table 5:** Compositions of optimized gluten‐free bread formulations containing whole chia flour

Trial	Component proportion in flour/starch blends^a^			Proximate composition (g/100 g)^b^					
	x_1_	x_2_	x_3_	Moisture	Ash	Lipid	Protein	Dietary fiber	Available carbohydrates^c^
A	0.95	0.00	0.05	52.11^a^ ± 0.05	1.25^c^ ± 0.01	4.65^c^ ± 0.11	5.37^c^ ± 0.06	2.81^b^ ± 0.16	33.81
B	0.90	0.00	0.10	52.17^a^ ± 0.10	1.30^b^ ± 0.01	5.13^b^ ± 0.10	5.91^b^ ± 0.08	3.43^a^ ± 0.24	32.07
C	0.86	0.00	0.14	52.20^a^ ± 0.06	1.36^a^ ± 0.01	5.62^a^ ± 0.06	6.27^a^ ± 0.02	3.94^a^ ± 0.28	30.61

a x_1_= Rice flour, x_2_= Potato starch, x_3_= Whole chia flour_._

b Values are means ± standard deviations (*n* = 3) and are expressed on g/100 g of food as eaten. Values followed by different superscripts in each row are significantly different (*p *<* *.05).

c Available carbohydrates were calculated by difference [100 – (moisture + ash + protein + fat + fiber)].

## 4 CONCLUSION

The application of a mixture design allowed finding that it is possible to add up to 14% WCF to an RF‐based GFB formulation while negligibly diminishing the loaf volume, crumb firmness and crumb moisture. The best formulations were prepared from RF blends with 5, 10 and 14% WCF, and they received overall acceptability scores similar to those of their white GFB and standard wheat bread counterparts. Incorporating 5%–14% whole chia flour in the formulation increased the levels of ash, lipid, protein and dietary fiber compared to those of the white GFB.

This research highlights the potential of WCF for producing nutrient‐dense and acceptable GFB, which is important for consumers with gluten‐related disorders because those products often lack nutrition content and acceptability.

## CONFLICTS OF INTEREST

The authors declare that they have no conflicts of interest.
